# Deoxyelephantopin Suppresses Pancreatic Cancer Progression *In Vitro* and *In Vivo* by Targeting linc00511/miR-370-5p/p21 Promoter Axis

**DOI:** 10.1155/2022/3855462

**Published:** 2022-06-25

**Authors:** Daolin Ji, Li Hou, Chunyang Xie, Haonan Feng, Dongdong Bao, Yue Teng, Junhao Liu, Tiangang Cui, Xiuhong Wang, Yi Xu, Gang Tan

**Affiliations:** ^1^Department of Hepatopancreatobiliary Surgery, The Fourth Affiliated Hospital, Harbin Medical University, Harbin, China; ^2^The Key Laboratory of Myocardial Ischemia, Harbin Medical University, Ministry of Education, Harbin, China; ^3^Department of Biochemistry and Molecular Biology, Harbin Medical University, Harbin, China; ^4^Department of Hepatopancreatobiliary Surgery, The Second Affiliated Hospital, Harbin Medical University, Harbin, China; ^5^Department of Pathology, Li Ka Shing Faculty of Medicine, The University of Hong Kong, Hong Kong

## Abstract

**Objectives:**

Deoxyelephantopin (DET) is a kind of natural active ingredient extracted from the Chinese herbal medicine *Elephantopus scaber* L. Many studies have revealed the potential antitumor effect on multiple malignancies. However, the detailed mechanism of its antitumor effect in pancreatic cancer remains unclear. Recently, studies have confirmed that noncoding RNA (ncRNA) plays an important regulatory role in malignancies. This research was performed to explore the relationship between ncRNA and DET-induced tumor inhibition in pancreatic cancer.

**Methods:**

Microarray profiling was applied to identify the candidate ncRNAs associated with DET-induced tumor inhibition. Quantitative real-time PCR was used to evaluate the expression of linc00511 in pancreatic cancer cells and tissues. The influence of DET on the cell proliferation, migration, and invasion was assessed by CCK-8, colony formation, wound healing, and Transwell assays. The relationship between lncRNAs, miRNAs, and p21 promoter region was analyzed by bioinformatics and verified by luciferase reporter gene and western blotting. The effect of linc00511 on nuclear translocation of miR-370-5p was explored by cytoplasmic and nuclear RNA purification. Moreover, the effect of DET on tumor growth and metastasis, and the prophylactic effect were investigated by establishing subcutaneous and lung metastatic tumor models.

**Results:**

Microarray assay indicated linc00511 was a potential target gene. The antitumor effect of DET in pancreatic cancer depended on downregulating linc00511 expression, and linc00511 might be an oncogene in pancreatic cancer. Silencing linc00511 enhanced the antitumor function of DET; conversely, linc00511 overexpression antagonized the DET cytotoxic effect. Additionally, miR-370-5p could bind to p21 promoter to exert the RNA activation and then promote p21 expression. *P*21 was a downstream gene of linc00511 and associated with pancreatic cancer progression. Linc00511 regulated p21 expression by blocking miR-370-5p nuclear translocation.

**Conclusions:**

To sum up, the present finding confirmed that DET suppressed the malignant biological behavior of pancreatic cancer via linc00511/miR-370-5p/p21 promoter axis.

## 1. Introduction

Pancreatic cancer is a kind of gastrointestinal malignancy with frustrating prognosis, 90% of which originates from the pancreas ductal epithelium [1, 2]. In recent years, although considerable advances in medical technology have been achieved, there is still an upward trend in the incidence and mortality of pancreatic cancer worldwide [3]. According to the realistic estimation data of GLOBOCAN 2020, pancreatic cancer ranks the seventh leading cause of cancer-related death between male and female groups, and will be pushed to the third leading cause of cancer-related death by 2025 due to its poor prognosis [4]. As described in the reports of American Cancer Society (ACS) and Cancer Research UK (CRUK), pancreatic cancer ranks the third leading cause of cancer-related death in the United States and the tenth most common malignancy in the UK, respectively [5, 6]. Additionally, the epidemiological investigation confirmed that during the last 10 years, among the cancer-related deaths in China, the proportion of deaths caused by pancreatic cancer has increased by about 9%, and the diets change and the aging of the population will make it worse [7]. Surgical resection is an effective treatment strategy for pancreatic cancer; however, due to its insidious onset and rapid progression rate, most pancreatic cancer patients have been already in the stage of local advanced or distant metastasis at the time of exact diagnosis, resulting in a dispiriting prognosis, and the five-year survival rate was still less than 5% [8–10]. Cumulative investigations have indicated that aggressive and effective chemotherapy strategies could ameliorate the prognosis of patients who have lost the chance of radical resection [11, 12]. However, the curative effect of traditional chemotherapeutic agents applied in clinical practice was still limited by the adverse reactions and tricky chemoresistance. Therefore, it is an urgent need to seek novel pharmacotherapy that is more effective and less toxic for pancreatic cancer patients.

Noncoding RNA (ncRNA), which is transcribed from the genome, is a special kind of RNA molecules that do not code for proteins [13, 14]. Genetic studies have shown that ncRNA could play a crucial regulatory role at the transcriptional and posttranscriptional levels, thus modulating the gene polymorphism and affecting the progression of multiple human diseases including malignancies [15]. Among them, long noncoding RNA (lncRNA) is a special type of ncRNA molecules with transcript length larger than 200 nt [16–18]. With the development of tumor genomics, the interaction between the aberrant expression of lncRNAs and tumor drew the researchers' attention. Multiple evidences indicated the abnormal expression of lncRNAs might lead to the occurrence and progression of various malignancies, including pancreatic cancer [19]. The mechanism of lncRNAs action is quite complex, including transcriptional interference, endogenous siRNA formation, and protein activity modulation. For example, Fu et al. [20] verified that the expression level of lncRNA HOTTIP was aberrantly upregulated in pancreatic cancer and affected the function of tumor stem cells through HOXA9; Wang et al. [21] reported that lncRNA CRNDE regulated the proliferation and metastasis abilities of pancreatic cancer cell by competing endogenous RNA (ceRNA) network sponging miR-384. In addition, it has also been confirmed that lncRNAs are in connection with chemoresistance of pancreatic cancer, such as PVT1, GAS5, and HOTAIR [22–24]. Many studies have suggested that changing the enrichment of oncogenic lncRNAs by external intervention could inhibit and even lead to the reversion of tumor malignant biological processes [25, 26]. It is believed that with rapid advances in sequencing technology and cancer informatics, more pancreatic cancer-related lncRNAs will be defined and applied as novel diagnostic or therapeutic targets.

Deoxyelephantopin (DET), a germacranolide sesquiterpene lactone, is a kind of natural active ingredient, which is extracted and purified from the Chinese herbal medicine *Elephantopus scaber* L. [27]. *Elephantopus scaber* L. has a wide range of pharmacological applications. In traditional Chinese medicine (TCM), it is used for treating a broad spectrum of common ailments, such as diarrhea, liver cirrhosis, rheumatism, and diabetes [27]. The rapid development of pharmacology has revealed the potential antitumor effect of DET on multiple malignancies, including colorectal carcinoma, hepatic carcinoma, breast cancer, and melanoma [28–30]. The effects of DET on malignant biological behavior and sensitivity of conventional chemotherapy drugs in pancreatic cancer were reported in our previous research uniquely [31]. It was verified that DET could induce cancer cell apoptosis through reactive oxygen species-dependent mitochondrial apoptosis pathway and alleviate the gemcitabine chemoresistance by modulating NF-*κ*B activation [31]. Nevertheless, the detailed mechanism of DET inhibiting the proliferation and metastasis abilities of pancreatic cancer is still not fully understood. Therefore, the present research is carried out to explore the advantageous antiproliferation and antimetastasis mechanism of DET on pancreatic cancer cells. High-throughput sequencing technology, various tumor cell lines, and different kind of experimental animal models were designed and applied to investigate the lncRNAs-related regulatory networks of DET in pancreatic cancer. In addition, considering that DET is used to treat diabetes in TCM, diabetes is a high-risk factor of pancreatic cancer, and the unique prophylactic effect of DET on pancreatic cancer was also studied. We hope that these explorations will improve the detection and treatment strategies for pancreatic cancer and provide an innovation of research method for TCM in tumor therapy.

## 2. Materials and Methods

### 2.1. Cell Lines Culture and Drug Preparation

Human pancreatic cancer cell lines (BxPC-3, CFPAC-1, PANC-1, SW1990, and MIAPaCa-2) and the corresponding control normal pancreatic ductal epithelial cells (HPDE6-C7) were commercially purchased from the Cell Line Resource Center, Chinese Academy of Life Sciences (Shanghai, China), and BeNa Biotechnology Research Institute (BNCC, Beijing, China), respectively. The cells mentioned above were maintained in RPMI-1640 or DMEM culture medium (Sigma-Aldrich, Shanghai, China) added 10% FBS (ABW, Shanghai, China) and 1% cell culture grade penicillin-streptomycin mixture (Gibco, Shanghai, China) under conventional culture conditions (37°C, 5% CO_2_). The cell applications were approved by the Ethics Committee of Harbin Medical University. Deoxyelephantopin (HPLC ≥98%) was bought from BioBioPha Co., Ltd. (Kunming, China) and dissolved in cell culture grade DMSO following the previous protocol [31].

### 2.2. Clinicopathological Tissue Samples

Excisional pancreatic cancer tissue samples and the corresponding paired normal pancreatic tissue samples of 91 patients were jointly collected from the Second and the Fourth Affiliated Hospital of Harbin Medical University. The written informed consent was obtained from all patients involved in the present study. Additionally, this operation also obtained approval from the Ethics Board of Harbin Medical University. After resection, all the tissues were immediately saved in liquid nitrogen until experimental use to avoid RNA or protein degradation.

### 2.3. Cell Transfection

The small interfering RNAs (siRNAs) that specifically target to silence linc00511 (si-linc00511-1 and si-linc00511-2), miRNAs mimics, double-stranded RNAs (dsRNAs), biotin-labeled miRNAs, and the corresponding controls were structured by GenePharma (Suzhou, China). These RNAs were transfected into cells using Lipofectamine 3000 (Invitrogen, CA, USA). Then, the cells were harvested and applied to experiments after transfection for 24, 48, and 72 h. Similarly, the pcDNA3.1 vector (GenePharma, Suzhou, China) targeting linc00511 was performed for stable aberrant expression of linc00511, and the transfection procedure was also conducted using Lipofectamine 3000.

### 2.4. RNA Preparation and qRT-PCR Assays

The total RNA samples from the cells and tissues were separated and obtained using TRIzol (Sigma-Aldrich, MO, USA). Furthermore, the RNA belonging to the nucleus or cytoplasm fraction was isolated by Cytoplasmic and Nuclear RNA Purification Kit (Norgen Biotek, ON, CA). RNA quantitative analysis was conducted using SYBR™ Green qPCR Master Mix Kit (Thermo Scientific, NY, USA) on CFX96 PCR System (Bio-Rad, CA, USA). In the present study, the relative change of target gene expression level in pancreatic cancer cells was calculated with 2^−ΔΔCt^ formula. In tissue samples, the target gene expression change was evaluated by standard ΔΔCt value. The primer sequences applied in the present research are listed in Supplementary Table 1.

### 2.5. Microarray Assay

Total RNA was extracted from general BxPC-3 cells and DET-treated cells (BxPC-3_D), and the difference of lncRNA expression spectrum between two groups was recognized using the Arraystar Human LncRNA Array V4.0 Analysis Platform (MD, USA). With the application of more advanced sequencing technique, more than 40,173 lncRNAs could be analyzed simultaneously. In our present research, the hybridization procedure was performed following the supplier's technique recommendations. In brief, the high-throughput sequencing mainly included the following steps: the cell total RNA extraction, RNA sample purification, complementary RNA (cRNA) transcription, complementary DNA (cDNA) labeling (including the Cy3-dCTP labeling and the Cy5-dCTP labeling), and cDNA hybridization to the analysis array (chip format used in present experiment is: 8 × 60 K, ≥ 2 probes for each kind of lncRNA). Finally, the high-throughput sequencing results were analyzed by hierarchical clustering; in addition, the lncRNAs identified for further study should simultaneously matched the following conditions: the expression level >2-fold change and *P* value < 0.05.

### 2.6. Cell Proliferation Assays

Cell viability is closely related to proliferation; therefore, the cell proliferation was initially evaluated by applying the Cell Counting Kit-8 (CCK-8, Dojindo, Shanghai, China). The transfected cells were collected and inoculated into 96-well culture plate with the density of 3 × 10^3^ cells per well. After the conditional intervention, 10 *μ*L of CCK-8 working solution was added into each well. After incubation for 2.5 h at 37°C without of light, the absorbance value at 450 nm was detected by Varioskan LUX multimode microplate reader (Thermo Scientific, NY, USA).

Moreover, Ki-67 immunofluorescence (IF) assay was performed. Briefly, tumor cells were pre-inoculated on sterile slides, and after DET intervention or transfection, 4% paraformaldehyde was added for cell immobilization. Next, the membrane permeability process was achieved by 0.1% Triton X-100 (22–24°C, 20 min), and the cells were incubated with Ki-67 antibody (1 : 200, Immunoway, Suzhou, China) overnight. Then, the cells were stained with a secondary antibody labeled with green fluorophore (1 : 1000, Abcam, Shanghai, China) for 1 h at 22–25°C. After the nuclei were stained with DAPI for 5 min, the fluorescence images were obtained under fluorescence microscope.

To further assess the change in cell proliferation, colony formation assays were performed. Pancreatic cancer cells after transfection or DET stimulation were seeded into 6-well culture plate (800 cells per well). After 14 days of ordinary culture, the macroscopical colonies (>50 cells) were fixed, stained, and counted manually.

### 2.7. Cell Migration and Invasion Assays

The mobility of tumor cells after transfection or DET stimulation was initially tested using wound healing assays. Briefly, cells were collected and inoculated into 6-well culture plate, and a micropipette tip (200 *μ*L) was applied to scrape a longitudinal cell-free area on the center of Petri dish when the cells were at high confluence level (>80%). Next, the floating cells were rinsed with bacteria-free PBS. Finally, serum-free medium was added, and the wound closure condition was observed under inverted microscope (Leica, Wetzlar, Germany) at special time points.

Transwell assays were carried out to further evaluate the cell migration. Briefly, cells with transfection or DET treatment were collected and resuspended in medium without FBS. Then, 200 *μ*L cell suspension containing about 6 × 10^4^ cells was transferred to the Transwell upper unit (8 *μ*m aperture, Jet Bio-Filtration, Guangzhou, China), and 700 *μ*L medium containing 10% FBS was added to the lower unit as chemical attractant. After 24 h of culture, the cells still stayed in the upper unit were wiped out using swab. Then, the cells penetrated through the semipermeable membrane were immobilized and stained. The translocated cells were photographed and counted under microscope. Additionally, the upper units pretreated with Matrigel (Corning, NY, USA) were applied to assess cell invasion.

### 2.8. *In vivo* Tumorigenicity Assays

The *in vivo* animal experiments were in accordance with the institutional guiding principles of the Animal Health Care and Use Committee of Harbin Medical University and approved by the Ethics Committee (ethical review number: SYDW2020-059). The BALB/c-nu mice (aged 6 weeks, 18–20 g weight) were commercially purchased from Vital River Laboratories (Beijing, China) and kept in individual ventilated cages (IVC) added with water and food that met specific pathogen-free (SPF) level. To evaluate the effects of transfection and drug intervention on tumor growth, 200 *μ*L of cell suspension (containing about 5 × 10^6^ cells) was subcutaneously injected into the right armpit. The intervention methods were similar to our previous study ^31^. The subcutaneous tumor size was observed and calculated every three days. The lung metastatic model was performed to analyze the effects of transfection and drug intervention on metastasis. Briefly, 200 *μ*L of transfected BxPC-3 cell suspension (containing about 3 × 10^6^ cells) was injected into the mouse caudal vein. After 6 weeks of feed and observation, the nude mice were sacrificed by spinal cord disconnection. The metastasis was assessed by the metastatic nodules number on the dissected lung specimens. In addition, to examine the preventive effect of DET on pancreatic cancer, the subcutaneous tumor model in nude mice was constructed again. The nude mice aged 4–5 weeks were selected and pre-treated with DET for 2 weeks before subcutaneous inoculation of tumor cells to mimic the prophylactic application scenario. The dose and frequency of drug administration were consistent with the above experiments.

### 2.9. Protein Extraction and Western Blot Assays

The total protein from pancreatic cancer cells was extracted and purified using RIPA lysis buffer added phenylmethanesulfonyl fluoride (100 : 1, Beyotime, Shanghai, China) and phosphatase inhibitor (100 : 3, Beyotime, Shanghai, China), and the concentration of total protein sample was quantitatively analyzed by applying BCA Protein Assay Kit (Abcam, Shanghai, China). After mixing with loading buffer (4 : 1, Beyotime, Shanghai, China), the protein samples were denatured by metal bath at 100°C for 5 min. Protein samples were divided using SDS-PAGE (concentration of 8%–12%; EpiZyme, Shanghai, China), and the separated-protein band was transferred to PVDF membranes (aperture of 0.22 or 0.45 *μ*m, Sigma-Aldrich, MO, USA). Then, the membranes containing separated proteins were co-incubated with different primary antibodies against GAPDH (1 : 10,000, Abcam, Shanghai, China), p21 (1 : 1000, Abcam, Shanghai, China), Snail (1 : 1000, Abcam, Shanghai, China), or ZEB1 (1 : 500, Abcam, Shanghai, China) in refrigerator at 4°C overnight. Next, the membranes were rinsed with TBST buffer and further incubated with secondary antibody (HRP-conjugated; Zsbio, Beijing, China) for 1.5 h at 20–22°C. Finally, target protein bands were imaged using ECL Kit (Yeasen, Shanghai, China) under Image Analysis System (Tanon, Shanghai, China). Different target protein expression levels were analyzed by ImageJ software (V1.52, NIH, MD, USA).

### 2.10. Dual-Luciferase Reporter Assays

The sequences of linc00511 incorporating the wild-type and mutant-type binding sites of target miRNAs were synthesized and cloned into the pmirGLO dual-luciferase reporter vector to construct the WT-linc00511-pmirGLO and MUT-linc00511-pmirGLO according to the manufacturer's instructions (Promega, WI, USA). Next, the WT/MUT-linc00511-pmirGLO was cotransfected into BxPC-3 or CFPAC-1 cells with target miRNA mimics or negative control mimics using Lipofectamine 3000 transfection reagent. After transfected for 48 h, the signal activities of Renilla and firefly luciferase were detected by applying Dual-Luciferase Report Gene Assay System (Promega, WI, USA).

### 2.11. Chromatin Immunoprecipitation (ChIP) Assays

After transfected the BxPC-3 and CFPAC-1 cells with biotin-labeled miRNAs, the ChIP assay (Sigma-Aldrich, Shanghai, China) was carried out in accordance with the supplier's manual. Briefly, 4 × 10^6^ cells were collected and prepared for each experiment; meanwhile, ribonuclease (RNase) inhibitors (Thermo Scientific, NY, USA) were applied to prevent miRNAs degradation. The cross-linking procedure of target chromatins was accomplished by 1% formaldehyde in a 37°C incubator for 15 min. Then, the immobilized cells were rinsed twice and resuspended by precooling SDS lysis buffer. Next, the mixture was further lysed by ultrasonic cracking apparatus (Thermo Scientific, NY, USA) with 2-mm miniature probe (frequency: 3–4 times, 10 s each time, output power: 30%) on ice. The chromatins in supernatant were prepurified with Protein *A* + *G* Agarose/Salmon Sperm DNA and incubated with biotin or IgG antibodies (Santa Cruz, TX, USA) at 4°C for 12 h. Finally, the antigen-antibody-DNA complex was collected and rinsed again. After purification and concentration procedures, the target gene was analyzed by qRT-PCR.

### 2.12. RNA Immunoprecipitation (RIP) Assays

In order to explore the interaction between lncRNAs and miRNAs, the RIP assays were carried out by applying the Magna RIP™ RNA-Binding Protein Immunoprecipitation Kit (Millipore, MA, USA). After transfection, the cells were collected and lysed by the special RIP lysis buffer. Immediately following lysis procedure, the cytolysate was cocultured with the magnetic beads conjugated with anti-Ago2 or anti-IgG antibodies. Finally, the enriched target ncRNA was quantified by qRT-PCR.

### 2.13. Histological Examination and Immunohistochemistry

The subcutaneous tumor tissues and lung specimens collected from the xenograft models were immediately immersed in 4% paraformaldehyde fixative. The fixed samples were then encased in paraffin (Sigma-Aldrich, Shanghai, China), sectioned to 4–5 *μ*m thin slices, and implemented to hematoxylin-eosin (HE) staining. In immunohistochemical staining, the samples were underwent deparaffinization and hydration, then followed with permeabilization using 0.5% Triton X-100 solution (Legend, Beijing, China) for 10 min. Next, the slices were blocked with 10% normal goat serum (Phygene, Fuzhou, China) for 20 min and incubated with primary antibody Ki-67 (1 : 800, Abcam, Shanghai, China), PCNA (1 : 5000, Cell Signaling Technology, MA, USA), or E-cadherin (1 : 500, Abcam, Shanghai, China) overnight in a 4°C refrigerator. After incubated with second antibody at 22–25°C for 1.5 h, the staining magnitude was recorded by photomicroscope (Carl Zeiss, Oberkochen, Germany).

### 2.14. Statistical Analysis

All experimental data involved in present study were represented by mean values ± standard deviation (SD). Statistical analysis was carried out through SPSS Statistics 27 software (IBM, NY, USA). The differences between two or multiple experimental groups were verified by applying one-way ANOVA analysis or Student's *t*-test. The *P* value < 0.05 was defined as statistically significant and labeled using different symbols. The prognosis between low or high linc00511 expression groups was exhibited by Kaplan–Meier curves using the log-rank test. All the experiments mentioned above were independently repeated at least triple times.

## 3. Results

### 3.1. The Change of lncRNAs Profiling in DET-Treated Pancreatic Cancer Cells

**Figure 1:**
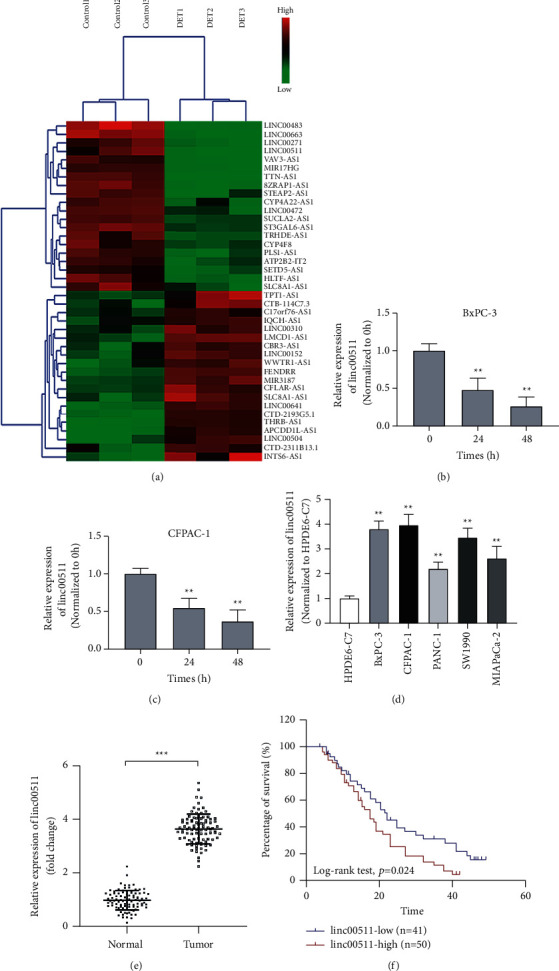
The heterogeneity of linc00511 profiling in pancreatic cancer cells and tissues, and linc00511 characterization. (a) Heat map and hierarchical clustering dendrogram of tumor-associated noncoding RNAs in BxPC-3 cells pre-treated with or without DET. The red color patches indicated high-expression level, and the green color patches indicated low-expression level. (b-c) The expression level of linc00511 after DET pretreatment in BxPC-3 and CFPAC-1 cells was analyzed by qRT-PCR. (d) The difference in expression level of linc00511 between human normal pancreatic ductal epithelial cells and pancreatic cancer cells was detected by qRT-PCR. (e) qRT-PCR analysis for the expression level of linc00511 in pancreatic cancer tissues and adjacent nontumor tissues. (f) Kaplan–Meier curve showed the overall survival of patients with low or high linc00511 expression. DET, deoxyelephantopin. All experiments were performed at least three times. Data were expressed as mean ± SD; ^*∗∗*^*P* < 0.01, ^*∗∗∗*^*P* < 0.001.

The antitumor effects of DET on pancreatic cancer cells had been expounded in our previous research [31]. However, the detailed antitumor mechanism had not been fully revealed. In our present research, the unique expression spectrum of lncRNAs between normal pancreatic cancer cells and DET-treated cells was detected by microarray assay. The candidate lncRNAs associated with DET-mediated tumor inhibition were explored, and the potential molecular mechanism behind the antitumor effect was revealed. A total of 241 differentially expressed lncRNAs were identified between the negative nondrug control group and the DET intervention group. Among the expression level of these lncRNAs being analyzed, 156 were consistently downregulated and the other 85 were consistently upregulated. As the hierarchical clustering analysis shown in Figure 1(a), the 40 different kinds of lncRNAs with the most significantly differential expression (20 with the most downregulated expression and 20 with the most upregulated expression) are listed. In combination with high-throughput sequencing data and documentation, linc00511, with the maximum fold change of expression level after DET stimulation, was identified and further studied. Next, the expression level of linc00511 in DET-treated BxPC-3 or CFPAC-1 cells was similarly analyzed by qRT-PCR. The results indicated that DET could decrease linc00511 expression level in a time-dependent pattern (Figures 1(b) and 1(c)).

### 3.2. Linc00511 was Highly Expressed in Pancreatic Cancer Cells and Tissues

The expression level of linc00511 between human normal pancreatic ductal epithelial cells and pancreatic cancer cells was examined by qRT-PCR. The expression of linc00511 was significantly higher in pancreatic cancer cells (Figure 1(d)). Similarly, as shown in Figure 1(e), the expression of linc00511 in pancreatic cancer tissues was also confirmed to be higher than that in the paired pericarcinomatous tissues. Next, with the median expression level of linc00511 as the critical value, 91 pancreatic cancer patients were assigned to two different groups, including the low-expression group or the high-expression group, respectively. As the Kaplan–Meier survival curve analysis shown in Figure 1(f), the overall survival of patients in the linc00511 low-expression group was obviously better than that in the linc00511 high-expression group. In summary, the above experimental results indicated that linc00511 might be an oncogene in pancreatic cancer and that the inhibitory effect of DET on tumor progression might be achieved through remodeling linc00511 expression.

### 3.3. Silencing linc00511 Facilitated DET-Mediated Tumor Inhibition *In Vitro*

**Figure 2:**
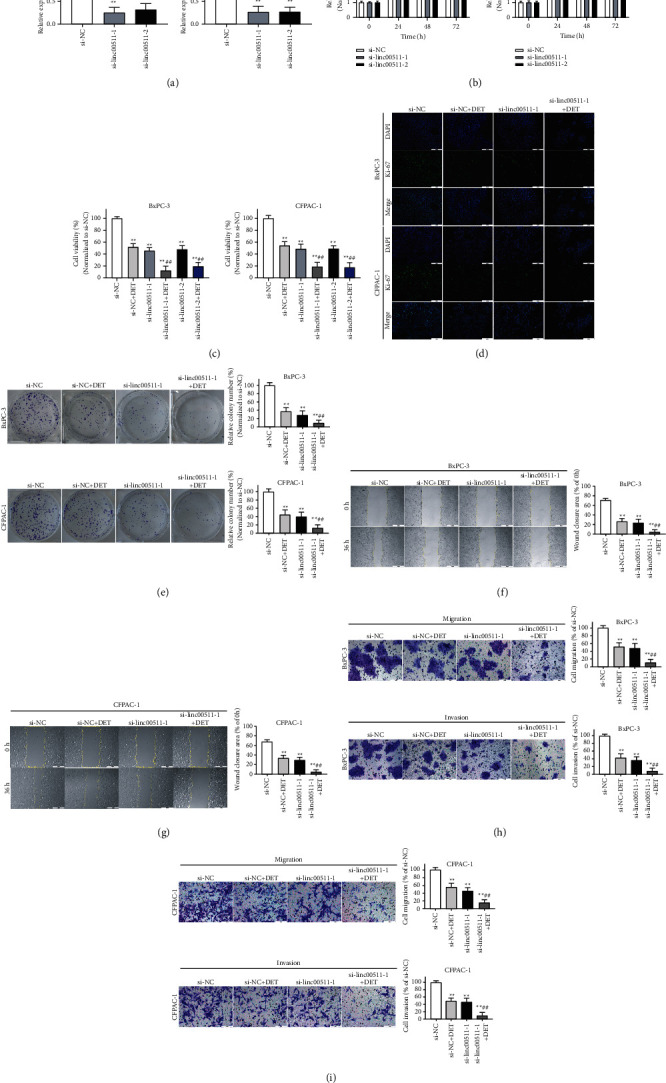
Silence of linc00511 enhanced the inhibitory effect of DET on tumor cell proliferation, migration, and invasion. (a) BxPC-3 and CFPAC-1 cells were transfected with si-NC or specific siRNAs targeting linc00511, and the knockdown efficiency was measured by qRT-PCR. (b) The cell viability of BxPC-3 and CFPAC-1 cells after siRNAs transfection was evaluated by CCK-8 assays. (c) The effect of DET treatment combined siRNAs transfection on cell viability was also detected by CCK-8 assays. (d) The location and expression level of Ki-67 in BxPC-3 and CFPAC-1 cells after silencing linc00511 or treating with DET were examined by IF. (e) The proliferation of BxPC-3 and CFPAC-1 cells after DET treatment, siRNAs transfection, or combined stimulation was assessed by colony formation experiments. (f-g) The mobility of BxPC-3 and CFPAC-1 cells after different stimulation was examined by wound healing assays. (h-i) The migration and invasion abilities of BxPC-3 and CFPAC-1 cells after different stimulation were assessed by Transwell assays. ^*∗∗*^*P* < 0.01 versus si-NC group. si-NC, siRNA negative control. ##*P* < 0.01 versus si-NC combined DET group. Magnification, × 40 (f, g), ×200 (d, h, i). Scale bar, 500 *μ*m (f, g), 100 *μ*m (d, h, i). DET, deoxyelephantopin. IF, immunofluorescence.

In order to elucidate the potential function of linc00511 on DET-mediated tumor inhibition, two different siRNAs targeting linc00511 (si-linc00511-1 and si-linc00511-2) were applied to silence linc00511 expression (Figure 2(a)). The cell viabilities of BxPC-3 and CFPAC-1 were suppressed by siRNAs transfection at the time point of 72 h (Figure 2(b)). In addition, the inhibitory effect of DET on pancreatic cancer cell viability was significantly enhanced by linc00511 siRNAs transfection, compared with that in the negative control group (Figure 2(c)). Considering the approximate silence efficiency of the two linc00511 siRNAs, si-linc00511-1 was selected for following experiments. Immunofluorescence assays found that DET and linc00511-siRNA mediated downregulation of linc00511 resulted in lower cell viability and Ki-67 fluorescence intensity, and this trend was enhanced by multimodal treatment (Figure 2(d)). Furthermore, the colony formation experiments were utilized to examine the proliferation ability of BxPC-3 and CFPAC-1 cells after DET treatment and siRNA transfection. As shown in Figure 2(e), compared with the DET or si-linc00511-1 mono-treatment group, the cell proliferation ability in DET and si-linc00511-1 combined group was significantly downregulated. Similar results were obtained in the wound healing and Transwell assays that were the cell migration and invasion abilities in DET and si-linc00511-1 combined group and were obviously downregulated compared to the DET or si-linc00511-1 mono-treatment group (Figures 2(f)–2(i)). All these results implied that silencing linc00511 expression enhanced the antiproliferation and antimetastasis effects of DET.

### 3.4. Linc00511 Overexpression Antagonized the Inhibitory Effect of DET *In Vitro*

**Figure 3:**
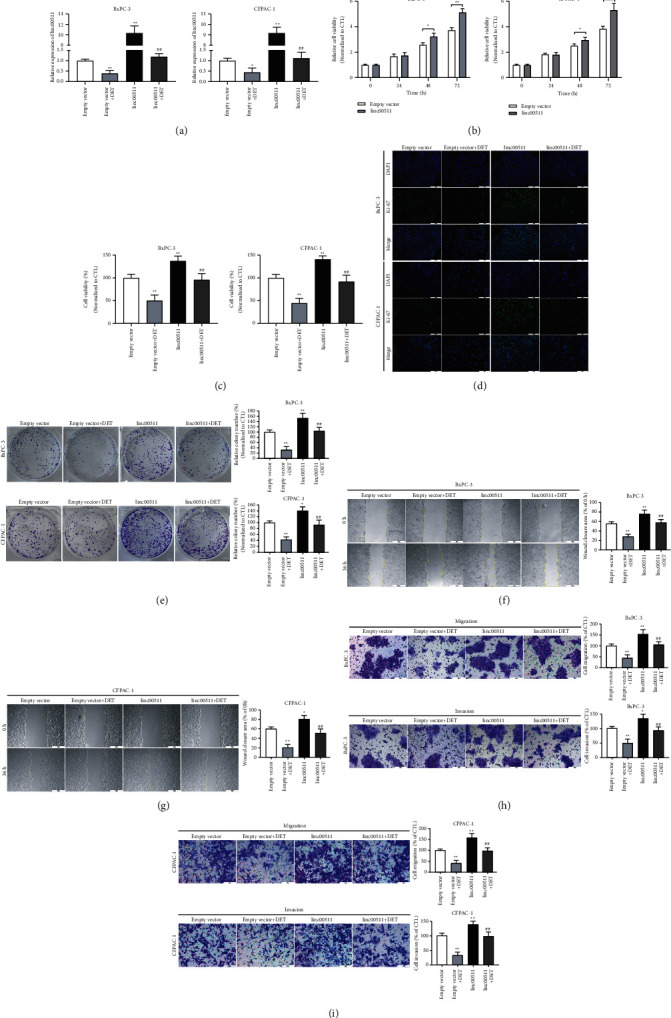
Linc00511 overexpression attenuated the suppressive effect of DET on tumor cell proliferation, migration, and invasion (a) BxPC-3 and CFPAC-1 cells were transfected with pcDNA3.1-linc00511 or corresponding empty vector, and the overexpression efficiency was analyzed by qRT-PCR. (b) The effect of linc00511 overexpression on cell viability was assessed by CCK-8 assays. (c) The effect of DET treatment combined pcDNA3.1-linc00511 transfection on cell viability was similarly detected by CCK-8 assays. (d) The location and expression of Ki-67 in cells after linc00511 overexpression or DET treatment were examined by IF. (e) The proliferation of BxPC-3 and CFPAC-1 cells after DET treatment, pcDNA3.1 transfection, or combined treatment was evaluated by colony formation assays. (f-g) The mobility of BxPC-3 and CFPAC-1 cells after different stimulation was estimated by wound healing assays. (h-i) The cell migration and invasion abilities after different treatment were detected by Transwell assays. ^*∗*^*P* < 0.05, ^*∗∗*^*P* < 0.01 versus empty vector group. ##*P* < 0.01 versus DET combined empty vector group. Magnification, × 40 (f, g), × 200 (d, h, i). Scale bar, 500 *μ*m (f, g), 100 *μ*m (d, h, i). DET, deoxyelephantopin. IF, immunofluorescence.

In the present research, the overexpression of linc00511 was achieved by pcDNA3.1-linc00511 transfection; in the meantime, the transfection efficiency was analyzed. As shown in Figure 3(a), pcDNA3.1-linc00511 transfection could effectively upregulate the linc00511 expression level and reverse the decreased expression of linc00511 induced by DET. Firstly, the overexpression of linc00511 could facilitate the proliferation ability of tumor cells, as shown in Figure 3(b). Next, the regulatory role of linc00511 was further explored by evaluating the proliferation and metastasis capacities. Compared to the control group, the DET-induced downregulation of cell viability was rescued by linc00511 overexpression (Figure 3(c)). Moreover, pcDNA3.1-linc00511 transfection-mediated upregulation of linc00511 leaded to the higher Ki-67 fluorescence signal intensity and antagonized the downward trend induced by DET treatment (Figure 3(d)). In BxPC-3 and CFPAC-1 cells, the linc00511 overexpression resulted to a conspicuous reversion on DET-mediated antiproliferation effect (Figure 3(e)). Furthermore, the DET-induced cell mobility downregulation was rescued by linc00511 overexpression (Figures 3(f) and 3(g)). The similar results were observed in Transwell assays, and lin00511 overexpression could counteract the inhibitory effect of DET on cell migration and invasion abilities (Figures 3(h) and 3(i)). To sum up, these data suggested that linc00511 played an important role in DET-mediated inhibitory action on tumorigenesis and further confirmed that linc00511 might act as an oncogene in pancreatic cancer.

### 3.5. Linc00511 Overexpression Attenuated the Antitumor Effect of DET in Pancreatic Cancer *In Vivo*

**Figure 4:**
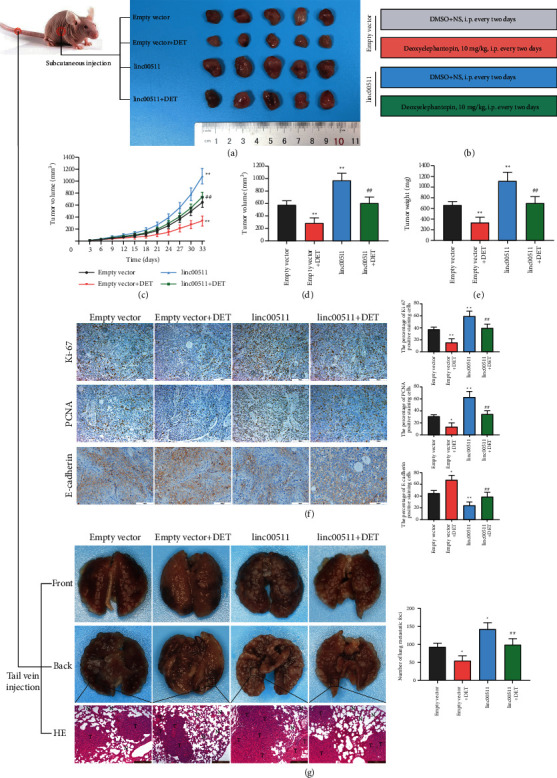
The effect of linc00511 on tumorigenesis and DET sensitivity *in vivo*. (a) Xenograft tumor model was obtained by subcutaneous injection of BxPC-3 cells transfected with pcDNA3.1-linc00511 or empty vector. (*n* = 5). (b) The detailed grouping strategies and intervention measures of xenograft model. (c) The curves of tumor volume in nude mice were plotted using measurements obtained every 3 days. (d) After 33 days of subcutaneous injection, complete resections were performed to obtain the tumors from the nude mice, and the more precise final tumor volume was assessed. (e) The final tumor weight was measured. (f) IHC staining targeting Ki-67, PCNA, and E-cadherin was carried out; meanwhile, the corresponding quantitative statistics were shown. (g) Lung metastatic tumor model was established by tail vein injection, and the HE staining was performed to identify the histopathological changes. (*n* = 4). ^*∗*^*P* < 0.05, ^*∗∗*^*P* < 0.01 versus empty vector group. ##*P* < 0.01 versus DET combined empty vector group. Magnification, × 200 (f), × 100 (g). Scale bar, 100 *μ*m (f), 200 *μ*m (g). IHC, immunohistochemistry. HE, hematoxylin and eosin.

In order to illuminate the role of linc00511 in pancreatic cancer progression and DET-mediated antitumor effect, the subcutaneous xenotransplanted tumor model of human pancreatic cancer in nude mice was designed. As the experiment data shown in Figure 4(a), linc00511 overexpression could reduce the drug sensitivity of pancreatic cancer to DET *in vivo.* The detailed grouping and intervention strategies are listed in Figure 4(b). Meanwhile, compared with that in the control group transfected with empty vector, the tumor growth rate in linc00511 overexpression group was significantly enhanced, as the growth curves and graphics shown in Figure 4(c). The similar tendency was obtained in the final tumor volume and weight analysis (Figures 4(d) and 4(e)). Additionally, the immunohistochemistry assay was performed to detect the expression level of proliferation-related markers (Ki-67 and PCNA) and epithelial-mesenchymal transition (EMT)-related marker (E-cadherin). As shown in Figure 4(f), the Ki-67 and PCNA expression levels in tumor samples of linc00511 overexpression or combined with DET treatment groups were higher than those in their respective control groups (empty vector group or DET single treatment group). By contrast, the level of E-cadherin was lower.

Next, the lung metastatic tumor model was performed to further investigate the special functional mechanism of linc00511 in DET-mediated antimetastatic effect *in vivo*. The specific grouping and treatment methods were consistent with the above (Figure 4(b)). After feeding and observing for 42 days, the experimental nude mice were sacrificed, and the lung specimens were collected. As the anterior and posterior images of lung specimens exhibited in Figure 4(g), linc00511 overexpression significantly enhanced the metastasis potential and antagonized the anti-metastasis effect of DET *in vivo*. In the meantime, to make this result more intuitive, the pathological histology analysis based on HE staining was implemented. The experimental data suggested that the normal alveolar structure was more severely damaged in linc00511 overexpression or DET combination groups, compared with the negative control or DET monotherapy group (Figure 4(g)). Taken together, these data confirmed that linc00511 was actually an oncogene in pancreatic cancer and linc00511 overexpression showed obvious antagonism to tumor inhibitory effect of DET.

### 3.6. DET Possessed Potential Preventive Effect on Pancreatic Cancer Progression In *Vivo*

A large number of studies have confirmed that diabetes mellitus is a non-negligible risk factor in pancreatic cancer. Moreover, the effective tumor inhibitory function in pancreatic cancer and high drug safety has been verified in our previous study [31]. Considering the *Elephantopus scaber* L., which is the source of DET, is applied to treat diabetes in TCM classics, the fundamental potential of DET for pancreatic cancer prevention was validated. The grouping and therapeutic strategies are listed in Figures S1(a) and S1(b). As the tumor growth curve shown in Figure S1(c), the tumor growth rate in DET pretreatment group was suppressed, compared with that in the control group. Additionally, the terminal tumor burden in DET pretreatment group was also lower (Figures S1(d) and S1(e)). Moreover, the IHC assays also indicated that DET pretreatment downregulated the Ki-67 expression and upregulated the E-cadherin expression (Figure S1(f)). Given the above, DET indeed holds a unique prophylactic effect *in vivo*.

### 3.7. DET-Induced Remodeling Expression of linc00511 Influenced the Expression Level of p21, Snail, and ZEB1, and Then Regulated the Proliferation and Metastasis Abilities of Pancreatic Cancer

**Figure 5:**
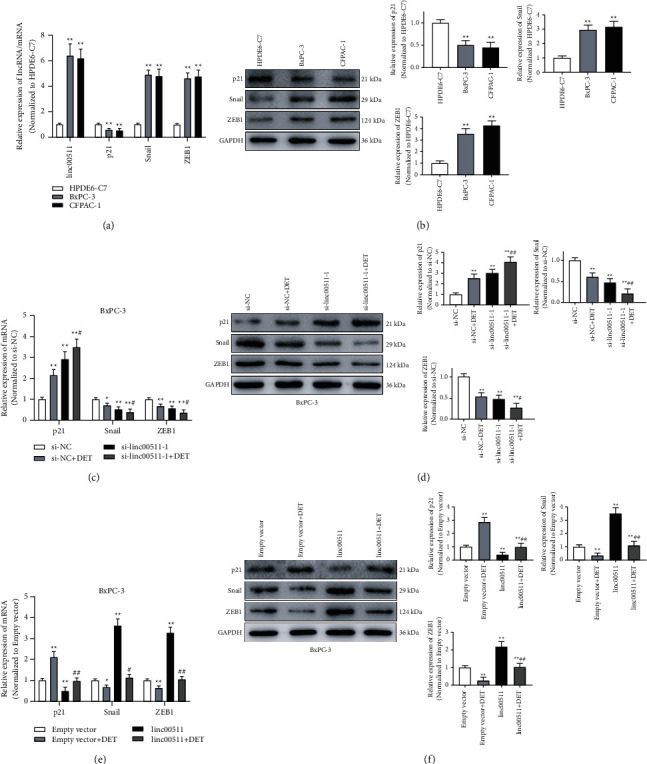
The differential expression of linc00511, p21, Snail, and ZEB1 between HPDE6-C7 cells and pancreatic cancer cells. (a) The expression levels of linc00511 and mRNA of p21, Snail, and ZEB1 were analyzed by qRT-PCR. ^*∗∗*^*P* < 0.01 versus HPDE6-C7 cells. (b) The immunoblotting assays and corresponding quantitative statistics were performed to evaluate the p21, Snail, and ZEB1 protein expression levels in HPDE6-C7, BxPC-3, and CFPAC-1 cells. ^*∗∗*^*P* < 0.01 versus HPDE6-C7 group. (c) The mRNA expression levels of p21, Snail, and ZEB1 of BxPC-3 cells after DET treatment, siRNA transfection, or combined stimulation were estimated by qRT-PCR. ^*∗*^*P* < 0.05, ^*∗∗*^*P* < 0.01 versus si-NC group. #*P* < 0.05 versus DET combined si-NC group. (d) The immunoblotting assays and corresponding quantitative statistics were performed to evaluate the p21, Snail, and ZEB1 levels affected by DET treatment or siRNA transfection. ^*∗∗*^*P* < 0.01 versus si-NC group. #*P* < 0.05, ##*P* < 0.01 versus DET combined si-NC group. (e) The mRNA expression levels of p21, Snail, and ZEB1 of BxPC-3 cells after DET treatment, pcDNA3.1-linc00511 transfection, or combined stimulation were estimated by qRT-PCR. ^*∗*^*P* < 0.05, ^*∗∗*^*P* < 0.01 versus empty vector group. #*P* < 0.05, ##*P* < 0.01 versus DET combined empty vector group. (f) The immunoblotting assays and corresponding quantitative statistics were carried out to assess the p21, Snail, and ZEB1 levels affected by DET or pcDNA3.1. ^*∗∗*^*P* < 0.01 versus empty vector group. ##*P* < 0.01 versus DET combined empty vector group.

Cumulative studies have reported that p21 is closely associated with TGF-*β*-mediated proliferation in tumor cells [32]. In addition, p21 could also regulate the expression of EMT-related proteins and affect the metastasis of malignancies [32]. So, in the present study, the qRT-PCR and western blotting assays were applied to examine the differential expression level of linc00511, p21, Snail, and ZEB1 between HPDE6-C7 and pancreatic cancer cells. As shown in Figures 5(a) and 5(b), the expression level of linc00511, Snail, and ZEB1 was higher in BxPC-3 and CFPAC-1 cells than these in HPDE6-C7 cells; on the contrary, the expression level of p21 was obviously lower. In order to reveal the linc00511 function in DET-mediated proliferation and metastasis inhibition, the mRNA level of p21, Snail, and ZEB1 in DET combined si-linc00511-1 treatment pancreatic cancer cells was tested by qRT-PCR. The experimental data suggested that DET treatment or si-linc00511-1 transfection could upregulate the expression of p21, and, meanwhile, downregulate the expression of Snail and ZEB1 compared with that of the control group (Figure 5(c)). Additionally, this tendency was enhanced by DET and si-linc00511-1 combination treatment group (Figure 5(c)). Similar protein expression levels were obtained in western blotting assay (Figure 5(d)). To further confirm the function of linc00511, experiments were carried out again in BxPC-3 cells to be treated with DET or pcDNA3.1-linc00511. As shown in Figure 5(e), compared with the control group and DET single treatment group, linc00511 overexpression by pcDNA3.1 transfection could significantly downregulate the mRNA expression of p21 and upregulate the mRNA expression of Snail and ZEB1. Moreover, pcDNA3.1-linc00511 transfection could also alleviate the DET-induced p21, Snail, and ZEB1 mRNAs expression change. Next, it was verified again in immunoblotting assay, and the same results were observed (Figure 5(f)). Combined with the above experimental results in Figures 2–4, it could be confirmed that DET-mediated proliferation and metastasis inhibitory effects in pancreatic cancer cells were achieved by regulating linc00511 and downstream target genes including p21, Snail, and ZEB1.

### 3.8. Identification of the Subcellular Localization of linc00511 in BxPC-3 and CFPAC-1 Cells

**Figure 6:**
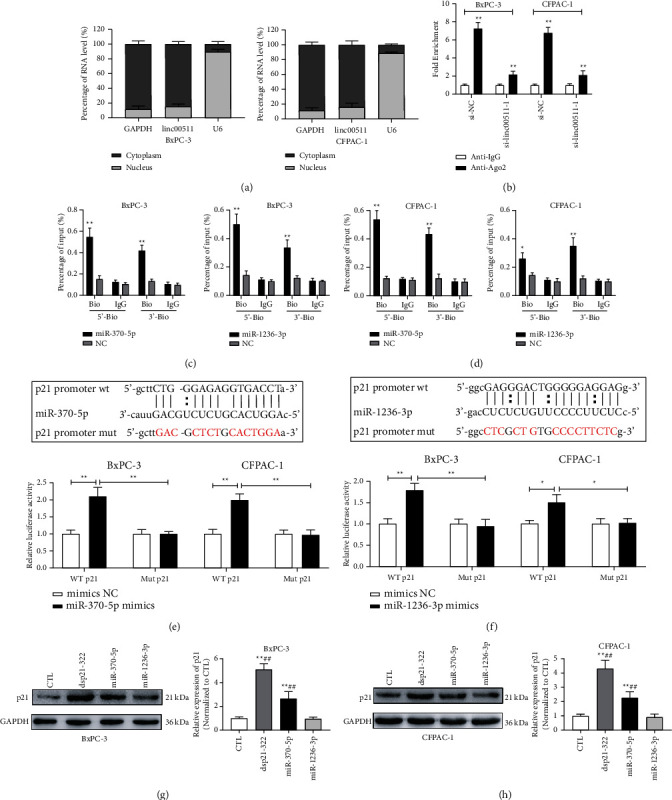
Subcellular localization of linc00511 and identification of miRNAs interacting with the p21 promoter region. (a) Relative levels of linc00511 in the nuclear and cytoplasmic parts of BxPC-3 and CFPAC-1 cells were evaluated by nuclear and cytoplasmic separation kit combined qRT-PCR. (b) Ago2 RNA-RIP assays for linc00511 enrichment in transfected BxPC-3 and CFPAC-1 cells. ^*∗∗*^*P* < 0.01. (c-d) ChIP assays were performed to evaluate the enrichment of predicted miRNAs at the corresponding target region of p21 promoter in BxPC-3 and CFPAC-1 cells. ^*∗*^*P* < 0.05, ^*∗∗*^*P* < 0.01 versus NC group. NC, negative control. (e-f) The schematic diagram of p21 promoter-wt and p21 promoter-mut luciferase reporter vectors; the binding potential between p21 promoter region and predicted miRNAs was assessed by dual-luciferase reporter assays in BxPC-3 and CFPAC-1 cells. ^*∗*^*P* < 0.05, ^*∗∗*^*P* < 0.01. (g-h) The p21 expression levels of BxPC-3 and CFPAC-1 cells after transfected with dsControl, dsp21, miR-370-5p, or miR-1236-3p were detected by immunoblotting assays. ^*∗∗*^*P* < 0.01 versus CTL group. CTL, control. ##*P* < 0.01 versus miR-1236-3p transfection group.

Based on the reported studies, the subcellular localization of lncRNA is closely associated with its regulatory mechanisms. As a consequence, the detailed localization of linc00511 in BxPC-3 and CFPAC-1 cells was detected by applying cytoplasmic and nuclear RNA purification experiment. As the systematic analysis shown in Figure 6(a), the linc00511 was predominantly enriched in the cytoplasm, compared to the nucleus, in pancreatic cancer BxPC-3 and CFPAC-1 cells. In addition, it was found that compared with that of anti-IgG group, linc00511 was significantly enriched in the anti-Ago2 immunoprecipitation group; instead, this procedure was attenuated by linc00511 knockdown (Figure 6(b)). Combined with the research status of lncRNA regulatory mechanism, this result implied that linc00511 might act as miRNA sponge to modulate the gene expression, furthermore, regulating the malignant biological behavior of pancreatic cancer cells.

### 3.9. MiRNAs Regulated the Activity of p21 Gene by Targeting the p21 Promoter Region in Pancreatic Cancer Cells

Previous studies had verified that some miRNAs could bind to the promoter region of p21 and thus affecting p21 activity [33]. Additionally, in our present research, it was confirmed that the expression trend between intracellular linc00511 and p21 protein was negatively correlated, and linc00511 could act as miRNAs sponge. Therefore, the target miRNAs, which could bind to the p21 promoter and connect with linc00511, were selected by bioinformatics analysis, including miR-370-5p and miR-1236-3p. Initially, the ChIP assay was applied to verify the binding potential of predicted miRNAs to the p21 promoter region. As shown in Figures 6(c) and 6(d), the DNA sequence of p21 promoter region could be pulled down more effectively by both the biotin-labeled miR-370-5p and miR-1236-3p, compared with that in the negative control group. Next, the interaction between these two miRNAs and p21 promoter region was further examined by applying dual-luciferase reporter assays. The luciferase reporter gene including the wild type of p21 promoter region sequence (wt-p21) or mutant type of p21 promoter region sequence (mut-p21) was established. As shown in Figures 6(e) and 6(f), miR-370-5p and miR-1236-3p mimics could significantly increase the fluorescence signal intensity from the cells, which transfected with wt-p21 promoter sequence; meanwhile, the cells transfected with mut-p21 promoter sequence showed almost constant luciferase activity. To further determine whether both miR-370-5p and miR-1236-3p could target the p21 promoter and upregulate p21 gene expression in BxPC-3 and CFAPC-1 cells, dsRNA, dsControl RNA, miR-370-5p, or miR-1236-3p was transfected into BxPC-3 and CFPAC-1 cells. RT-qPCR and immunoblotting assays were applied to detect the p21 mRNA and protein level. The data indicated that only miR-370-5p could activate the p21 gene expression in both BxPC-3 and CFPAC-1 cells, compared with miR-1236-3p (Figures 6(g) and 6(h)). In summary, although both miR-370-5p and miR-1236-3p possessed the binding potential to p21 promoter region, only miR-370-5p exhibited the regulatory effect on p21 activity.

### 3.10. Linc00511 Acted as an RNA Sponge to Adsorb miR-370-5p and Affected the Translocation of miR-370-5p from Cytoplasm to Nucleus, Thereby Remodeling RNA Activation and Regulating the Expression of p21

**Figure 7:**
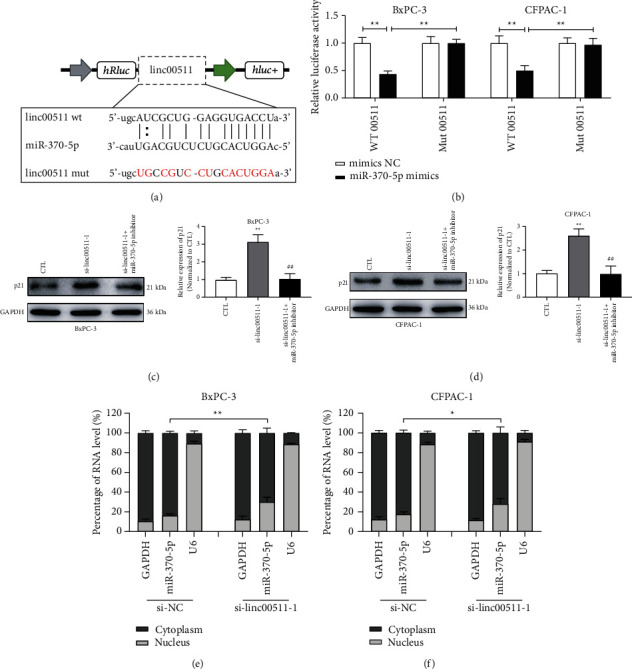
Linc00511 negatively regulated p21 activity by interacting with miR-370-5p in pancreatic cancer cells. (a-b) The schematic diagram of linc00511-wt and linc00511-mut luciferase reporter vectors; the binding potential between linc00511 and miR-370-5p was tested by dual-luciferase reporter assays in BxPC-3 and CFPAC-1 cells. ^*∗∗*^*P* < 0.01. (c-d) The p21 expression levels of BxPC-3 and CFPAC-1 cells after transfected with si-linc00511 or miR-370-5p inhibitor were detected by immunoblotting assays. ^*∗∗*^*P* < 0.01 versus CTL group. CTL, control. ^##^*P* < 0.01 versus si-linc00511-1 single transfection group. (e-f) The relative levels of miR-370-5p in the nuclear and cytoplasmic parts of BxPC-3 and CFPAC-1 cells transfected with si-NC or si-linc0511-1 were evaluated by nuclear and cytoplasmic purification kit combined qRT-PCR assays. ^*∗*^*P* < 0.05, ^*∗∗*^*P* < 0.01.

To explore the binding potential between linc00511 and miR-370-5p, the luciferase reporter gene containing wild type or mutant type of linc00511 sequence was designed and synthesized. As the experiment data shown in Figure 7(a), the luciferase activity of BxPC-3 or CFPAC-1 cells transfected with wt-linc00511 was downregulated by miR-370-5p mimics; on the contrary, the fluorescence signal intensity of cells transfected with mut-linc00511 was nearly unchanged (Figure 7(b)). Then, the si-linc00511-1 and miR-370-5p inhibitors were cotransfected into BxPC-3 and CFPAC-1 cells, and the immunoblotting assays were performed to detect the p21 expression level. Depletion of si-linc0511-1 promoted the p21 protein level; however, this effect was rescued by cotransfection with miR-370-5p inhibitor (Figures 7(c) and 7(d)). In order to further validate the effect of linc00511 on nucleus translocation of miR-370-5p, the cytoplasmic and nuclear RNA purification assays were performed in BxPC-3 and CFPAC-1 cells. Compared with that in the si-NC group, the expression of miR-370-5p in the nucleus was upregulated by si-linc00511-1 transfection (Figures 7(e) and 7(f)). All the data indicated that linc00511 might retard the nucleus translocation of miR-370-5p, remodel RNA activation, and modulate p21 expression level. In conclusion, the regulatory mechanism of DET on the proliferation and metastasis abilities of pancreatic cancer cells was potentially carried out through linc00511/miR-370-5p/p21 promoter axis.

## 4. Discussion

At present, pancreatic cancer still remains one of the most lethal malignancies of the digestive system worldwide [34]. In recent years, there have been a lot of substantial advances in medical technology, but the early diagnosis and therapeutic strategy of pancreatic cancer are still unsatisfied. Although the 5-year survival rate for patients receiving radical excision is still less than 25%, surgical method, such as pancreaticoduodenectomy or distal pancreatectomy, remains the only curative therapeutic strategy for pancreatic cancer patients [35]. Moreover, compared with the traditional open surgery, the advances in surgical methods including laparoscopic and robotic-assisted operations have improved the safety of surgical resection of pancreatic cancer [35]. However, given the worse biological characteristics of insidious onset and rapid progression, it is worth noting that more than 30% of patients with pancreatic cancer have been already in local advanced stage at the time of diagnosis; in the meantime, at least 50% of patients might suffer from distant metastasis [35]. For this part of patients who have lost the opportunity of surgical treatment, systematic chemotherapy is an essential weapon. Based on this important strategy, several types of chemotherapeutic agents targeting different pathways have been explored and developed to combat tumor progression, such as antimetabolites, alkylating agents, and immunosuppressants [36–38]. It should be warned that chemotherapy is a double-edged sword in the treatment of pancreatic cancer. Adverse drug reactions and chemoresistance during chemotherapy still should not be ignored. The main purpose of present research is to explore novel antitumor agents with higher drug safety to improve the prognosis status of pancreatic cancer.

The occurrence and development of malignancies involve extremely complex processes. Therefore, the application of single-target chemotherapeutic agent to treat tumors might not achieve expected efficacy. Recently, the novel concept of applying natural products, especially plant-derived natural compounds, to treat or even prevent malignant tumors has attracted a lot of attentions from researchers [39]. Three billion years of evolution and selection in nature have contribute to the diversity of natural compounds and the complexity of their molecular structures, making natural plants ideal candidates for the development of antitumor agents [40]. Based on this view, traditional Chinese medicine (TCM) provides a glimmer of hope in the therapeutic of malignancies. With the development of oncology and pharmacology, more and more TCM extracts have been proved to possess inhibitory effects on tumor, such as curcumin, quercetin, and evodiamine [41–43]. Deoxyelephantopin, a kind of sesquiterpene lactone compound, is derived from Chinese herbal medicine *Elephantopus scaber* L. [44]. Recently, the antitumor potential of DET has been explored in many common malignancies, but the detailed mechanism in pancreatic cancer is still uncovered. According to the TCM documents, *Elephantopus scaber* L. was applied to treat diabetes mellitus; in addition, diabetes has been verified to be a high-risk factor for pancreatic cancer, so the antitumor effect and underlying mechanism of DET in pancreatic cancer attracted the attention of our research team. In our previous research, it has been confirmed that DET could induce apoptosis by upregulating the ROS level and activating the caspase cascade reaction in pancreatic cancer and, meanwhile, improve the chemosensitivity of gemcitabine through modulating NF-*κ*B signaling pathway [31]. Moreover, in previous study, the reliable drug safety of DET was initially verified [31]. Nevertheless, the inhibitory potential of DET on proliferation, migration, and invasion of pancreatic cancer cells is still unclear. The purpose of our present study was to elucidate and solve this mystery.

Noncoding RNAs (ncRNAs) are defined as a special type of RNA molecules that do not have the ability to encode proteins [45]. A large number of studies indicated that ncRNAs could be artificially classified into several types broadly based on the structure and nucleotide length of the transcript, such as microRNAs (miRNAs) group (nucleotide length nearly 20–24 bp), long noncoding RNAs (lncRNAs) group (nucleotide length >200 bp) and circular RNAs (circRNAs) group (specially closed circular structure) [46]. Cumulative researches have indicated that ncRNAs could play regulatory role at different levels, including transcriptional level and posttranscriptional level, thus affecting the multiple normal biological procedures and disease progressions, including malignant tumors [46]. Therefore, a variety of ncRNAs have been discovered, and the relationship between them and different tumors has been extensively studied [47]. More importantly, these developments might be positively translated to clinical applications, such as diagnostic biomarkers, therapeutic targets, and prognostic assessments [47]. In our previous study, the antitumor effect of Huaier extract on human cholangiocarcinoma was revealed by analyzing lncRNAs expression profile [26]. We have got inspiration from the previous research strategy, and the high-throughput sequencing assay was applied to our present study. According to the sequencing data, the expression level of linc00511 was dramatically downregulated by DET, compared with the control group. Comprehensive analysis results suggested that the DET-mediated inhibition of proliferation and metastasis in pancreatic cancer cells was closely associated with the remodeling of linc00511. The antitumor effect of DET could be facilitated by linc00511 knockdown; on the contrary, it was antagonized by linc00511 overexpression. In addition, the oncogenic potential of linc00511 in pancreatic cancer was confirmed.

Previous studies have confirmed that the cyclin-dependent kinase (CDK) inhibitor p21, which is also named as p21^WAF1/Cip1^, is an essential member of the cyclin-dependent kinase inhibitor (CDKI) family [48]. It has been confirmed that p21 could affect the cell cycle by remodeling CDKs activity, thus regulating cell proliferation [48]. Compared with that in the corresponding paracancerous tissues, the expression level of p21 was lower in the cancer tissues [49–51]. Additionally, some recent studies have confirmed that p21 could also play a regulatory role in EMT procedure, therefore affecting tumor metastasis [52]. In breast cancer, Wang et al. [53] reported that p21 could suppress metastasis by modulating EMT-associated maker, such as Snail, E-cadherin, and ZEB1. In the meantime, sporadic studies have been reported in pancreatic cancer [52]. In our present study, the correlation between p21 expression level and DET-mediated inhibitory effect on proliferation and metastasis of pancreatic cancer was explored. Experimental data showed that the p21 level was lower in pancreatic cancer tissues than that in the corresponding pericarcinomatous tissues, and the same trend was observed in cell lines. Moreover, after DET stimulation, the p21 level was upregulated. In consideration of the DET-induced linc00511 expression downregulation, the regulatory mechanism between linc00511 and p21 expression was bewildering. Linc00511 knockdown upregulated the p21 expression; by contrast, linc00511 overexpression downregulated the p21 level. Combined with recent novel issues about RNA activation (RNAa) and miRNAs nuclear translocation, we found that some miRNAs could bind to p21 promoter region to upregulate p21 gene activity. In view of the abundant miRNAs binding sites of lncRNAs, we wondered that whether linc00511 regulated the p21 activation by targeting miRNAs, and the relationship between lncRNAs, miRNAs, and p21 promoter region was investigated. In our study, the miR-370-5p was confirmed to act as a mediator. In our present study, the result indicated that some miRNAs could bind to the p21 promoter region, but whether or not they play a role in RNA activation may differ from cell to cell, and the more detailed research should be carried out in the future. Additionally, there are still many issues that need to be uncovered; for example, whether the expression of miR-370-5p is regulated by linc00511, and whether the activity of miRNAs transport-related proteins is also modulated by DET. At present, there are few studies on the regulatory effect of drugs on nuclear translocation of ncRNAs and RNAa mechanism. In the future studies, we hope to further decipher the secret of this biological process, thus making contribution to tumor research.

## 5. Conclusions

To sum up, we have preliminary found that linc00511 blocked miR-370-5p nuclear translocation to bind p21 promoter region by sponge effect, thus remodeling RNA activation and p21 expression. It was indicated that the inhibitory effect of DET on proliferation and metastasis of pancreatic cancer cells was achieved by linc00511/miR-370-5p/p21 promoter region axis. Additionally, DET possesses a unique prophylactic effect on pancreatic cancer.

## Data Availability

The data used to elucidate the findings of this research are available from the corresponding author upon request.

## Supplementary Materials

Supplementary Materials Supplementary Table 1. The primer sequences applied in the present study. Supplementary Figure 1: the preventive effect of DET on pancreatic cancer *in vivo*. (a) The schematic diagram of detailed grouping strategies and intervention measures in xenograft model. (b) Xenograft tumor model was established by subcutaneous injection of BxPC-3 cells. (c) The curves of tumor volume in nude mice. (d) The final tumor volume was calculated. (e) The final tumor weight was examined. (f) IHC staining targeting Ki-67 and E-cadherin was carried out; meanwhile, the corresponding quantitative statistics were shown. ^*∗*^*P* < 0.05, ^*∗∗*^*P* < 0.01. Magnification, × 200 (*f*). Scale bar, 100 *μ*m (*f*). IHC, immunohistochemistry.

## References

[1] Moore A., Donahue T. (2019). Pancreatic cancer. *JAMA*.

[2] Saluja A., Maitra A. (2019). Pancreatitis and pancreatic cancer. *Gastroenterology*.

[3] Siegel R. L., Miller K. D., Jemal A. (2020). Cancer statistics, 2020. *CA: A Cancer Journal for Clinicians*.

[4] Sung H., Ferlay J., Siegel R. L. (2021). Global cancer statistics 2020: GLOBOCAN estimates of incidence and mortality worldwide for 36 cancers in 185 countries. *CA: A Cancer Journal of Clinicians*.

[5] Mizrahi J. D., Surana R., Valle J. W., Shroff R. T. (2020). Pancreatic cancer. *The Lancet*.

[6] Maisonneuve P. (2019). Epidemiology and burden of pancreatic cancer. *La Presse Médicale*.

[7] Zhao C., Gao F., Li Q., Liu Q., Lin X. (2019). The distributional characteristic and growing trend of pancreatic cancer in China. *Pancreas*.

[8] Kang M. J., Jang J. Y., Kim S. W. (2016). Surgical resection of pancreatic head cancer: what is the optimal extent of surgery?. *Cancer Letters*.

[9] Fonseca A. L., Fleming J. B. (2018). Surgery for pancreatic cancer: critical radiologic findings for clinical decision making. *Abdom Radiol (NY)*.

[10] Strobel O., Neoptolemos J., Jäger D., Büchler M. W. (2019). Optimizing the outcomes of pancreatic cancer surgery. *Nature Reviews Clinical Oncology*.

[11] Gupta R., Amanam I., Chung V. (2017). Current and future therapies for advanced pancreatic cancer. *Journal of Surgical Oncology*.

[12] Neoptolemos J. P., Kleeff J., Michl P., Costello E., Greenhalf W., Palmer D. H. (2018). Therapeutic developments in pancreatic cancer: current and future perspectives. *Nature Reviews Gastroenterology & Hepatology*.

[13] Hombach S., Kretz M. (2016). Non-coding RNAs: classification, Biology and functioning. *Advances in Experimental Medicine & Biology*.

[14] Panni S., Lovering R. C., Porras P., Orchard S. (2020). Non-coding RNA regulatory networks. *Biochimica et Biophysica Acta (BBA) - Gene Regulatory Mechanisms*.

[15] Anastasiadou E., Jacob L. S., Slack F. J. (2018). Non-coding RNA networks in cancer. *Nature Reviews Cancer*.

[16] Sanchez Calle A., Kawamura Y., Yamamoto Y., Takeshita F., Ochiya T. (2018). Emerging roles of long non-coding RNA in cancer. *Cancer Science*.

[17] Chi Y., Wang D., Wang J., Yu W., Yang J. (2019). Long non-coding RNA in the pathogenesis of cancers. *Cells*.

[18] Fang Y., Fullwood M. J. (2016). Roles, functions, and mechanisms of long non-coding RNAs in cancer. *Genomics, Proteomics & Bioinformatics*.

[19] Lv Y., Huang S. (2019). Role of non-coding RNA in pancreatic cancer. *Oncology Letters*.

[20] Fu Z., Chen C., Zhou Q. (2017). LncRNA HOTTIP modulates cancer stem cell properties in human pancreatic cancer by regulating HOXA9. *Cancer Letters*.

[21] Wang G., Pan J., Zhang L., Wei Y., Wang C. (2017). Long non-coding RNA CRNDE sponges miR-384 to promote proliferation and metastasis of pancreatic cancer cells through upregulating IRS1. *Cell Proliferation*.

[22] Zhou C., Yi C., Yi Y. (2020). LncRNA PVT1 promotes gemcitabine resistance of pancreatic cancer via activating Wnt/*β*-catenin and autophagy pathway through modulating the miR-619-5p/Pygo2 and miR-619-5p/ATG14 axes. *Molecular Cancer*.

[23] Liu B., Wu S., Ma J. (2018). lncRNA GAS5 reverses EMT and tumor stem cell-mediated gemcitabine resistance and metastasis by targeting miR-221/SOCS3 in pancreatic cancer. *Molecular Therapy - Nucleic Acids*.

[24] Wang L., Dong P., Wang W., Huang M., Tian B. (2017). Gemcitabine treatment causes resistance and malignancy of pancreatic cancer stem-like cells via induction of lncRNA HOTAIR. *Experimental and Therapeutic Medicine*.

[25] Renganathan A., Felley-Bosco E. (2017). Long noncoding RNAs in cancer and therapeutic potential. *Advances in Experimental Medicine & Biology*.

[26] Ji D., Zheng W., Huang P. (2020). Huaier restrains cholangiocarcinoma progression in vitro and in vivo through modulating lncRNA TP73-AS1 and inducing oxidative stress. *OncoTargets and Therapy*.

[27] Lagoutte R., Serba C., Winssinger N. (2018). Synthesis of deoxyelephantopin analogues. *Journal of Antibiotics*.

[28] Mehmood T., Maryam A., Ghramh H. A., Khan M., Ma T. (2017). Deoxyelephantopin and isodeoxyelephantopin as potential anticancer agents with effects on multiple signaling pathways. *Molecules*.

[29] Chao W. W., Cheng Y. W., Chen Y. R., Lee S. H., Chiou C. Y., Shyur L. F. (2019). Phyto-sesquiterpene lactone deoxyelephantopin and cisplatin synergistically suppress lung metastasis of B16 melanoma in mice with reduced nephrotoxicity. *Phytomedicine*.

[30] Gao W., Sun J., Wang F. (2019). Deoxyelephantopin suppresses hepatic stellate cells activation associated with inhibition of aerobic glycolysis via hedgehog pathway. *Biochemical and Biophysical Research Communications*.

[31] Ji D., Zhong X., Huang P. (2020). Deoxyelephantopin induces apoptosis via oxidative stress and enhances gemcitabine sensitivity in vitro and in vivo through targeting the NF-*κ*B signaling pathway in pancreatic cancer. *Aging (Albany NY)*.

[32] Zhu F., Dai S. N., Xu D. L. (2020). EFNB2 facilitates cell proliferation, migration, and invasion in pancreatic ductal adenocarcinoma via the p53/p21 pathway and EMT. *Biomedicine & Pharmacotherapy*.

[33] Wang C., Chen Z., Ge Q. (2014). Up-regulation of p21(WAF1/CIP1) by miRNAs and its implications in bladder cancer cells. *FEBS Letters*.

[34] Froeling F. E. M., Casolino R., Pea A., Biankin A. V., Chang D. K. (2021). Molecular subtyping and precision medicine for pancreatic cancer. *Journal of Clinical Medicine*.

[35] Torphy R. J., Fujiwara Y., Schulick R. D. (2020). Pancreatic cancer treatment: better, but a long way to go. *Surgery Today*.

[36] Adel N. (2019). Current treatment landscape and emerging therapies for pancreatic cancer. *American Journal of Managed Care*.

[37] Philip P. A., Lacy J., Portales F. (2020). Nab-paclitaxel plus gemcitabine in patients with locally advanced pancreatic cancer (LAPACT): a multicentre, open-label phase 2 study. *The Lancet Gastroenterology & Hepatology*.

[38] Foschini F., Napolitano F., Servetto A. (2020). FOLFIRINOX after first-line gemcitabine-based chemotherapy in advanced pancreatic cancer: a retrospective comparison with FOLFOX and FOLFIRI schedules. *Ther Adv Med Oncol*.

[39] Khan M., Maryam A., Qazi J. I., Ma T. (2015). Targeting apoptosis and multiple signaling pathways with icariside II in cancer cells. *International Journal of Biological Sciences*.

[40] Ogbourne S. M., Parsons P. G. (2014). The value of nature’s natural product library for the discovery of New Chemical Entities: the discovery of ingenol mebutate. *Fitoterapia*.

[41] Wan M., Tajuddin W. N. B., Lajis N. H., Abas F., Othman I., Naidu R. (2019). Mechanistic understanding of curcumin’s therapeutic effects in lung cancer. *Nutrients*.

[42] Reyes-Farias M., Carrasco-Pozo C. (2019). The anti-cancer effect of quercetin: molecular implications in cancer metabolism. *International Journal of Molecular Sciences*.

[43] Jiang Z. B., Huang J. M., Xie Y. J. (2020). Evodiamine suppresses non-small cell lung cancer by elevating CD8+ T cells and downregulating the MUC1-C/PD-L1 axis. *Journal of Experimental & Clinical Cancer Research*.

[44] Kabeer F. A., Rajalekshmi D. S., Nair M. S., Prathapan R. (2017). Corrigendum to “molecular mechanisms of anticancer activity of deoxyelephantopin in cancer cells”. *Integrative Medicine Research*.

[45] Slack F. J., Chinnaiyan A. M. (2019). The role of non-coding RNAs in oncology. *Cell*.

[46] Zhang P., Wu W., Chen Q., Chen M. (2019). Non-coding RNAs and their integrated networks. *Journal of Integrative Bioinformatics*.

[47] Wang W. T., Han C., Sun Y. M., Chen T. Q., Chen Y. Q. (2019). Noncoding RNAs in cancer therapy resistance and targeted drug development. *Journal of Hematology & Oncology*.

[48] Parveen A., Akash M. S. H., Rehman K., Kyunn W. W. (2016). Dual role of p21 in the progression of cancer and its treatment. *Critical Reviews in Eukaryotic Gene Expression*.

[49] Teramen H., Tsukuda K., Tanaka N. (2011). Aberrant methylation of p21 gene in lung cancer and malignant pleural mesothelioma. *Acta Medica Okayama*.

[50] Llanos S., García-Pedrero J. M., Morgado-Palacin L., Rodrigo J. P., Serrano M. (2016). Stabilization of p21 by mTORC1/4E-BP1 predicts clinical outcome of head and neck cancers. *Nature Communications*.

[51] Cao N., Yu Y., Zhu H. (2020). SETDB1 promotes the progression of colorectal cancer via epigenetically silencing p21 expression. *Cell Death & Disease*.

[52] Wu B. Q., Jiang Y., Zhu F., Sun D. L., He X. Z. (2017). Long noncoding RNA PVT1 promotes EMT and cell proliferation and migration through downregulating p21 in pancreatic cancer cells. *Technology in Cancer Research and Treatment*.

[53] Wang L., Wang R., Ye Z. (2018). PVT1 affects EMT and cell proliferation and migration via regulating p21 in triple-negative breast cancer cells cultured with mature adipogenic medium. *Acta Biochimica et Biophysica Sinica*.

