# Supplementary figures and images for: Deoxyelephantopin Suppresses Pancreatic Cancer Progression In Vitro and In Vivo by Targeting linc00511/miR-370-5p/p21 Promoter Axis
Source: J Oncol. 2022 Jun 25;2022:3855462. doi: 10.1155/2022/3855462 (PMC9252706; doi:10.1155/2022/3855462)

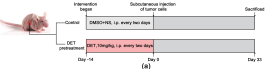

(a)

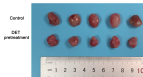

(b)

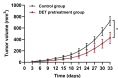

(c)

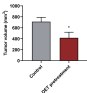

(d)

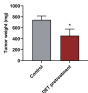

(e)

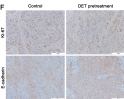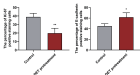

(f)

Supplement: Supplementary Materials — Supplementary Table 1. The primer sequences applied in the present study. Supplementary Figure 1: the preventive effect of DET on pancreatic cancer in vivo. (a) The schematic diagram of detailed grouping strategies and intervention measures in xenograft model. (b) Xenograft tumor model was established by subcutaneous injection of BxPC-3 cells. (c) The curves of tumor volume in nude mice. (d) The final tumor volume was calculated. (e) The final tumor weight was examined. (f) IHC staining targeting Ki-67 and E-cadherin was carried out; meanwhile, the corresponding quantitative statistics were shown. ∗P < 0.05, ∗∗P < 0.01. Magnification, × 200 (f). Scale bar, 100 μm (f). IHC, immunohistochemistry. [file 3855462.f1.zip › 3855462.f1/Supplementary Figure 1.pdf]
